# An Epithelial–Mesenchymal Transition–Driven Transcriptional Index Stratifies Immunosuppression and Therapeutic Resistance in Bone Malignancies

**DOI:** 10.1155/ijog/6315219

**Published:** 2026-06-04

**Authors:** Lihe Pang, Ye Hua, Zhaofei Chen, Guoya Wu

**Affiliations:** ^1^ Department of Joint Surgery, Xianning Central Hospital, The First Affiliated Hospital of Hubei University of Science and Technology, Xianning, Hubei, China

## Abstract

The aggressive clinical course and limited treatment avenues for key bone malignancies, specifically osteosarcoma, Ewing′s sarcoma, and giant cell tumor of bone, are heavily dictated by their intricate tumor microenvironment (TME). To decode this cellular heterogeneity and isolate viable prognostic markers, we synthesized single‐cell and bulk transcriptomic data across multiple bone cancer cohorts. Single‐cell profiling unveiled a complex TME hierarchy, while non–negative matrix factorization of the malignant compartment isolated a distinct transcriptional metaprogram heavily driven by the epithelial–mesenchymal transition (EMT). By extracting key genes modulating this EMT axis, we deployed CoxBoost and random survival forest modeling to distill an eight‐gene prognostic framework, designated the bone score. Across three independent patient cohorts, elevated bone scores consistently tracked with significantly diminished overall survival. Beyond serving as a survival metric, this signature mapped directly onto an immunosuppressive phenotype, characterized by depleted immune cell infiltration and blunted immune checkpoint signals, and predicted broad resistance to a panel of nine chemotherapeutic agents. Ultimately, this machine learning–derived index provides a refined, biologically grounded tool for risk stratification, capturing the tumor–stroma crosstalk that drives treatment failure in bone cancer.

## 1. Introduction

Primary bone malignancies, chiefly osteosarcoma, Ewing′s sarcoma, and giant cell tumor of bone, drive disproportionate mortality among pediatric and adolescent populations [1]. While localized disease is clinically manageable, prognosis remains poor for metastatic or relapsed cases, where the 5‐year overall survival rate remains below 30% despite intensified multimodal regimens [2]. This therapeutic ceiling is no longer viewed simply as a failure of conventional drugs, but rather as a failure to systematically decode the profound intratumoral heterogeneity and the protective tumor microenvironment (TME) that orchestrate treatment evasion.

At the core of this adaptive resistance and metastatic dissemination lies the epithelial–mesenchymal transition (EMT) axis [3, 4]. Although recent high‐resolution single‐cell RNA sequencing (scRNA‐seq) has begun to map the subclonal architecture of bone cancers, revealing disparate malignant subpopulations driven by divergent transcriptional networks, the specific clinical footprint of EMT‐driven metaprograms (MPs) remains elusive [5]. The field critically lacks actionable biomarkers capable of translating these complex, single‐cell‐derived EMT signatures into tangible clinical stratification.

To bridge this translational gap, we systematically integrated multicohort scRNA‐seq and bulk transcriptomic data to decrypt the cellular ecology of bone malignancies. By isolating a core EMT‐driven transcriptional MP exclusively within the malignant compartment, we engineered and validated a machine learning–derived prognostic framework: the bone score. Crucially, we demonstrate that this index extends far beyond conventional risk stratification. It robustly quantifies TME immunosuppression, predicts the likelihood of response to immunotherapies, and maps broad resistance profiles across multiple chemotherapeutic agents, thereby providing a highly calibrated tool to guide precision oncology in bone cancer [6, 7].

## 2. Materials and Methods

### 2.1. Data Collection and Processing

The scRNA‐seq datasets (GSE168664, GSE198896 [8], and GSE212341 [9]) for bone cancer (Ewing′s sarcoma, osteosarcoma, and giant cell tumor of bone) were downloaded from the GEO database. The bulk RNA sequencing dataset for osteosarcoma was collected from the TARGET database, as well as two independent GEO datasets (GSE21257 [10] and GSE39055 [11]).

### 2.2. Computational Analysis

The EMT‐related gene list was obtained from the Hallmark database [12]. The R package Seurat [13] was implemented to process the scRNA‐seq dataset. The R package InferCNV was used to define malignant cells. Normal cells were defined using canonical marker genes. The pathway enrichment analysis of GO and KEGG terms was conducted using the R package SCP. To investigate transcriptional heterogeneity among malignant cells, non–negative matrix factorization (NMF) was applied to the cancer cell expression matrix using the GeneNMF package [14, 15]. The optimal number of MPs was determined by assessing the cophenetic correlation coefficient and the residual sum of squares, which identified eight stable transcriptional MPs. Genes correlated with MP3 (*R* > 0.5) were extracted. Genes upregulated in malignant cells compared to other normal cells were identified. A machine learning algorithm, CoxBoost [16], was used for dimension reduction of prognostic genes. Random survival forest (RSF) [17] was further used to calculate the bone score. The correlation between the bone score and stromal score, immune score, ESTIMATE score, and tumor purity was evaluated using the ESTIMATE algorithm [6, 18]. The association between bone score and immune cell infiltration was predicted through the TIMER [19]. Nine immunotherapeutic signatures, including CYT [20], IFN*γ*IS [21], chemokineIS [22], RIR [23], ICBnetIS [7], AyersExpIS [21], RohIS [24], DavoliIS [19], and GEP [21], were used for immunotherapy prediction of bone score. The drug prediction of bone score was performed using the R package pRRophetic [25]. A *p* value of 0.05 or less indicated statistical significance.

## 3. Results

### 3.1. Single‐Cell Transcriptomic Landscape of Bone Cancer

**Figure 1:**
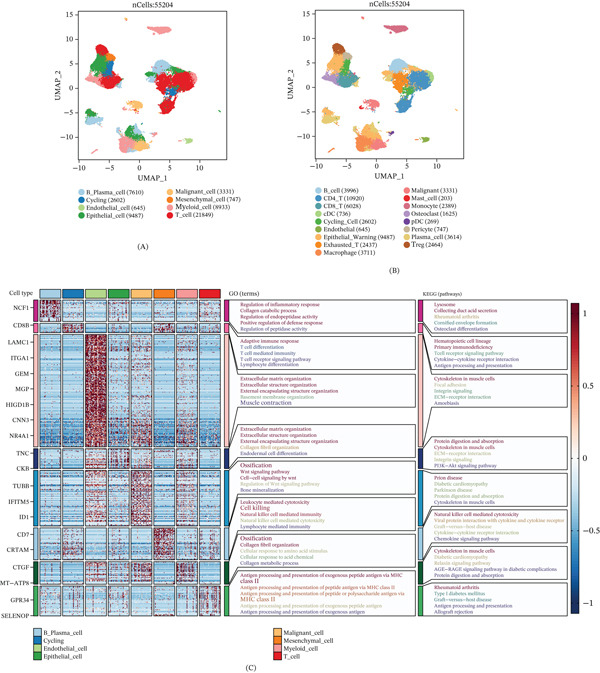
Exploration of bone cancer microenvironment at the scRNA‐seq level. (A) UMAP shows the major cell types. (B) The heatmap shows the marker gene and functional annotation of major cell types. (C) UMAP projection showing 17 annotated minor cell subpopulations within the bone cancer microenvironment.

We first analyzed three scRNA‐seq datasets comprising Ewing′s sarcoma, osteosarcoma, and giant cell tumor of bone samples. Unsupervised clustering identified eight major cell types, including B plasma cells, cycling cells, endothelial cells, epithelial cells, malignant cells, mesenchymal cells, myeloid cells, and T cells (Figure 1A). Further subclustering revealed 17 distinct minor cell populations, providing a high‐resolution atlas of bone cancer TME (Figure 1C).

### 3.2. Identification of EMT‐Related Transcriptional Program in Malignant Cells

**Figure 2:**
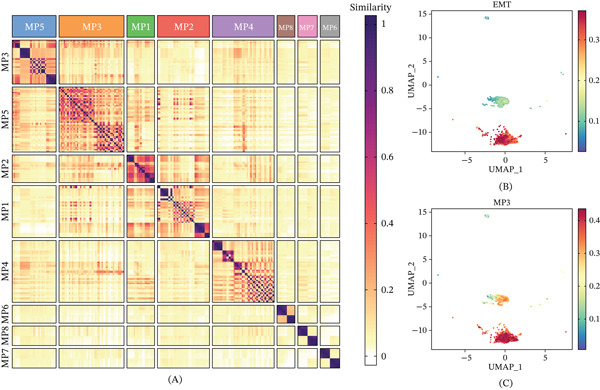
Identification of EMT‐related genes. (A) Heatmap showing Jaccard similarity coefficients for pairwise comparisons among eight robust NMF programs in cancer cells. (B) UMAP shows EMT scores across the malignant cell population. (C) UMAP shows the MP3 signature scores in cancer cells.

To investigate transcriptional heterogeneity among malignant cells, we performed NMF analysis on the malignant cell expression matrix. Eight stable transcriptional MPs were identified based on the cophenetic correlation coefficient and residual sum of squares (Figure 2A). Among these, MP3 showed the strongest correlation with EMT scores across the malignant cell population (Figure 2B,C), indicating that MP3 represents an EMT‐related transcriptional program in bone cancer.

### 3.3. Development and Validation of a Bone Score Prognostic Model

**Figure 3:**
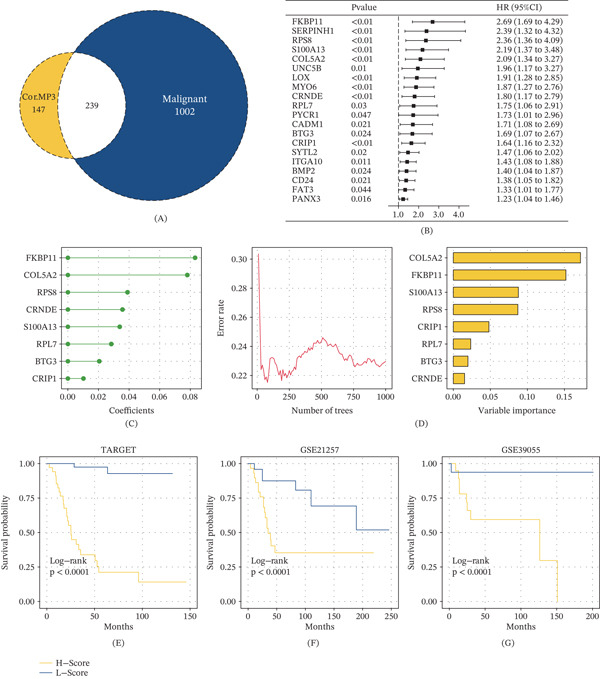
Development of a machine learning–based prognostic model. (A) A Venn diagram shows the intersection of genes correlated with MP3 signature scores and genes upregulated in malignant cells. (B) Univariate Cox regression analysis reveals the prognostic genes. (C) CoxBoost for dimension reduction of prognostic genes. (D) RSF for dimension reduction of prognostic genes. (E) Survival curves of bone score–based groups in the TARGET cohort. (F) Survival curves of bone score–based groups in the GSE21257 cohort. (G) Survival curves of bone score–based groups in the GSE39055 cohort.

We extracted 239 genes that were both correlated with MP3 (Spearman correlation coefficient > 0.5) and upregulated in malignant cells compared to normal cells (Figure 3A). Univariate Cox regression analysis identified 20 genes significantly associated with overall survival (Figure 3B). We then applied the CoxBoost algorithm for dimension reduction, which retained 10 prognostic genes (Figure 3C). Further refinement using the RSF algorithm identified eight key genes (*COL5A2*, *FKBP11*, *S100A13*, *RPS8*, *CRIP1*, *RPL7*, *BTG3*, and *CRNDE*), which we used to construct the bone score (Figure 3D).

To translate our single‐cell findings into a clinically testable format, we mapped this eight‐gene signature onto bulk RNA datasets. Survival analysis demonstrated that a high bone score was significantly associated with worse overall survival in the TARGET cohort (Figure 3E) and this association was further validated in two independent GEO cohorts (GSE21257 and GSE39055; Figure 3F,G). These results confirm that bone score is a robust prognostic biomarker for bone cancer.

### 3.4. Functional Annotation of Bone Score

**Figure 4:**
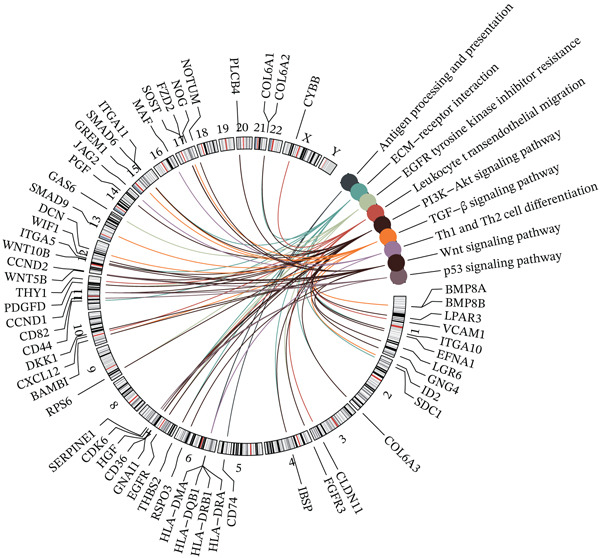
Functional annotation of bone score. KEGG pathway enrichment analysis on bone score–based DEGs and their distribution in human chromosomes.

KEGG pathway enrichment analysis of differentially expressed genes between high and low bone score groups revealed enrichment in multiple cancer‐related pathways, including antigen processing and presentation, ECM–receptor interaction, EGFR tyrosine kinase inhibitor resistance, leukocyte transendothelial migration, PI3K–Akt signaling pathway, TGF‐beta signaling pathway, Th1 and Th2 cell differentiation, Wnt signaling pathway, and p53 signaling pathway (Figure 4).

### 3.5. Bone Score Predicts Drug Sensitivity

**Figure 5:**
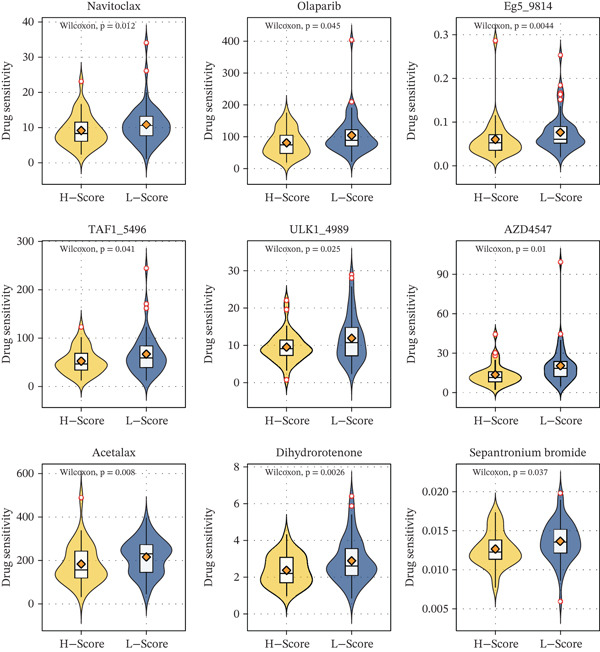
Drug prediction of bone score. Drug sensitivity of nine drugs in bone score–based groups.

We next performed an unbiased screening across the default drug panel in the pRRophetic package. High bone score was associated with decreased sensitivity to nine drugs, including navitoclax, olaparib, Eg5_9814, TAF1_5496, ULK1_4989, AZD4547, acetalax, dihydrorotenone, and sepantronium bromide (Figure 5). These findings suggest that patients with high bone scores may benefit less from these chemotherapeutic agents.

### 3.6. Bone Score Correlates With Immune Microenvironment Features

**Figure 6:**
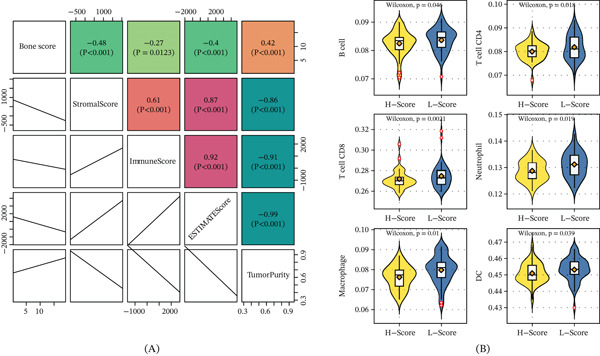
Immune features of bone score. (A) The correlation between bone score and microenvironment scores (stromal score, immune score, ESTIMTE score, and tumor purity). (B) The box plot shows the abundance of six immune cells in bone score–based groups.

ESTIMATE analysis showed that a high bone score was associated with lower stromal score, immune score, and ESTIMATE score, but higher tumor purity (Figure 6A). Consistently, TIMER analysis revealed significantly lower infiltration levels of B cells, CD4+ T cells, CD8+ T cells, neutrophils, macrophages, and dendritic cells in the high bone score group (Figure 6B).

### 3.7. Bone Score Predicts Immunotherapeutic Response

**Figure 7:**
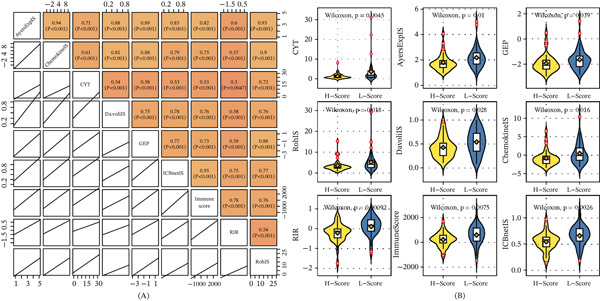
Immunotherapy prediction of bone score. (A) The heatmap shows the correlation between bone score and nine immunotherapeutic signatures. (B) The box plot shows the levels of nine immunotherapeutic signatures in bone score–based groups.

To assess the potential of bone score in predicting immunotherapy response, we analyzed nine established immunotherapeutic signatures. All nine signatures, including CYT, IFN*γ*IS, chemokineIS, RIR, ICBnetIS, AyersExpIS, RohIS, DavoliIS, and GEP, showed significantly lower scores in the high bone score group (Figure 7A,B). These results indicate that patients with high bone scores are less likely to respond to immune checkpoint blockade therapy.

## 4. Discussion

In this study, we provide a unified biological index to quantify the aggressive nature of bone malignancies. Rather than relying on single‐gene markers, we demonstrated that an EMT‐driven transcriptional MP deeply influences the bone cancer microenvironment, serving as a critical link between cellular heterogeneity, immune exclusion, and broad therapeutic resistance.

Our single‐cell analysis provides a high‐resolution atlas of bone cancer TME, confirming the presence of diverse cell populations that contribute to tumor progression and treatment resistance. The identification of an EMT‐related transcriptional program in malignant cells is consistent with previous studies showing that EMT drives bone cancer metastasis and drug resistance [26]. By focusing on genes associated with this program, we developed a prognostic model that captures the biological aggressiveness of bone cancer.

The functional enrichment analysis revealed that a high bone score is associated with multiple cancer‐related pathways, including PI3K–Akt [27], TGF‐beta [28], and Wnt [29] signaling pathways, which are known to play critical roles in bone cancer progression. Importantly, we found that a high bone score correlates with an immunosuppressive TME [30], characterized by reduced immune cell infiltration and lower expression of immunotherapeutic signatures. This finding is particularly significant, as it suggests that bone score could be used to identify patients unlikely to benefit from immune checkpoint blockade therapy, thereby avoiding unnecessary treatment and associated toxicities.

Furthermore, our drug sensitivity analysis identified nine chemotherapeutic agents to which high‐bone‐score patients are less sensitive. These results provide valuable insights for personalized chemotherapy selection. For example, patients with high bone score may require alternative treatment strategies, such as combination therapies targeting the enriched pathways identified in our study.

Our findings align with broader systemic evaluations of the TME across various solid tumors. Recent advances highlight the critical regulatory roles of EMT‐related chemokine axes in mediating systemic immune evasion, remodeling the microenvironment, and driving treatment resistance beyond the primary tumor site [31, 32]. Evaluating these systemic immune features provides vital context for understanding why local bone tumors so effectively resist therapy.

Our study has several limitations. First, a major limitation is that we combined three distinct bone malignancies (osteosarcoma, Ewing′s sarcoma, and giant cell tumor of bone) into a single analytical framework. While they share certain microenvironmental traits, their distinct cellular origins and genetic drivers mean our unified bone score may not perfectly capture disease‐specific nuances. Second, the prognostic model was developed and validated using retrospective public datasets, and prospective clinical trials are needed to confirm its clinical utility. Third, the functional mechanisms underlying the association between bone score and immune suppression require further experimental validation. Finally, our study focused on transcriptomic data, and integration with genomic and proteomic data may provide a more comprehensive understanding of bone cancer biology.

In conclusion, our EMT‐driven transcriptional index offers a biological lens to evaluate immune suppression in bone malignancies. While not yet ready for the clinic, this tool provides a foundation for future prospective studies aiming to stratify patient risk and explore alternative combination therapies for those unlikely to respond to standard care.

## Author Contributions

Lihe Pang: conceptualization, methodology, software, formal analysis, and writing original draft; Ye Hua: data curation, investigation, visualization, and writing original draft; Zhaofei Chen: conceptualization, resources, and supervision; Guoya Wu: project administration, validation, and supervision. Zhaofei Chen and Guoya Wu jointly supervised this work. Lihe Pang and Ye Hua contributed equally to this work.

## Funding

No funding was received for this manuscript.

## Consent

The authors have nothing to report.

## Conflicts of Interest

The authors declare no conflicts of interest.

## Data Availability

The single‐cell and bulk transcriptomic datasets analyzed during the current study are available in public repositories, including the Gene Expression Omnibus (GEO) database under Accession Numbers GSE168664, GSE198896, GSE212341, GSE21257, and GSE39055 and the TARGET database. All custom scripts, computational pipelines, and processed analytical data underlying the results reported in this manuscript are available from the corresponding authors upon reasonable request.
